# Familial history of hypertension-induced impairment on heart rate variability was not observed in strength-trained subjects

**DOI:** 10.1590/1414-431X20187310

**Published:** 2018-11-14

**Authors:** F.A. Santa-Rosa, G.L. Shimojo, M. Sartori, A.C. Rocha, J.V. Francica, J. Paiva, M.C. Irigoyen, K. De Angelis

**Affiliations:** 1 Laboratório de Fisiologia Translacional, Universidade Nove de Julho (UNINOVE), São Paulo, SP, Brasil; 2 Instituto do Coração, Faculdade de Medicina, Universidade de São Paulo, São Paulo, SP, Brasil; 3 Laboratório do Movimento Humano, Universidade São Judas Tadeu, São Paulo, SP, Brasil; 4 Departamento de Fisiologia, Universidade Federal de São Paulo, São Paulo, SP, Brasil

**Keywords:** Family history, Arterial blood pressure, Cardiac autonomic modulation, Sympathetic modulation, Strength training

## Abstract

Family history of hypertension is an important predictive factor for hypertension and is associated with hemodynamic and autonomic abnormalities. Previous studies reported that strength training might reduce arterial blood pressure (AP), as well as improve heart rate variability (HRV). However, the benefits of strength training in the offspring of hypertensive parents have not been fully evaluated. Here, we analyzed the impact of strength training on hemodynamics and autonomic parameters in offspring of hypertensive subjects. We performed a cross-sectional study with sedentary or physically active offspring of normotensives (S-ON and A-ON) or hypertensives (S-OH and A-OH). We recorded RR interval for analysis of HRV. AP was similar between groups. Sedentary offspring of hypertensives presented impairment of total variance of RR interval, as well as an increase in cardiac sympathovagal balance (S-OH: 4.2±0.7 *vs* S-ON: 2.8±0.4 and A-ON: 2.4±0.1). In contrast, the strength-trained group with a family history of hypertension did not show such dysfunctions. In conclusion, sedentary offspring of hypertensives, despite displaying no changes in AP, showed reduced HRV, reinforcing the hypothesis that autonomic dysfunctions have been associated with higher risk of hypertension onset. Our findings demonstrated that strength-trained offspring of hypertensives did not present impaired HRV, thus reinforcing the benefits of an active lifestyle in the prevention of early dysfunctions associated with the onset of hypertension in predisposed populations.

## Introduction

Hypertension is one of the major risk factors for development of cardiovascular disease (1). Currently, 25% of the world's population suffers from hypertension and it has been estimated that this figure will have risen by 60% by 2025, reaching a prevalence of 40% (2).

Family history is an important non-modifiable risk factor for hypertension onset (3,4). It is now well established that the autonomic nervous system (sympathetic and parasympathetic) is one of the determinants of arterial blood pressure. In fact, several studies have reported reduced heart rate variability (HRV) and/or increased sympathetic modulation/activation in offspring of hypertensives (3,5). Evidence suggests that the possibility of an inherited syndrome of abnormalities, regardless of high arterial blood pressure, may predispose this population for hypertension (6). In this sense, cardiac autonomic dysfunction may play a critical role in hypertension onset in offspring of hypertensives.

On the other hand, a physically active lifestyle has been recognized as a non-pharmacological strategy for the prevention of chronic diseases, such as hypertension, metabolic syndrome, and diabetes (7). Aerobic exercise training improves cardiovascular autonomic control and arterial blood pressure (8). Moreover, although the guidelines recommend strength exercises as a complementary tool to aerobic exercise (9), the cardiovascular and autonomic benefits of strength training remain unclear. Despite the benefits of strength training on cardiovascular health in humans (10) and in an experimental model (11), the benefits of strength training in offspring of hypertensives have yet to be fully understood. We hypothesized that strength training may be an alternative strategy for preventing the onset of hypertension. Here, we analyzed the impact of strength exercise training on hemodynamics and on HRV in offspring of hypertensives.

## Material and Methods

We performed a cross-sectional study with healthy offspring of normotensive or hypertensive parents. The study protocol was approved by the Ethics Committee of the Universidade São Judas Tadeu, and all subjects signed an informed consent form. Forty-four male offspring of normotensive or hypertensive parents were recruited using convenience sampling and assigned into four groups (n=11 in each group): sedentary offspring of normotensives (S-ON), sedentary offspring of hypertensives (S-OH); physically active offspring of normotensives (A-ON); and physically active offspring of hypertensives (A-OH).

Subjects who presented office arterial blood pressure (AP) lower than 140/90 mmHg on three different occasions were considered healthy normotensive, following the guidelines of the British Society of Hypertension. Offspring of hypertensives was defined as such when at least one of the parents was hypertensive. Subjects with concomitant diseases, under pharmacological treatment, or reporting cigarette smoking, heavy drinking, or food addiction were excluded from the sample. We also excluded subjects with an uncertain family history of hypertension or with parents or siblings who had secondary forms of hypertension or diabetes mellitus type 1 or 2.

In order to determine the level of physical activity we used the International Physical Activity Questionnaire (IPAQ, version 6). Twenty-two subjects with or without family history of hypertension (n=11 each group) that had at least 6 months of supervised practice of strength training on 3 non-consecutive days of the week at the time of the study recruitment were compared with twenty-two sedentary subjects with or without family history of hypertension (n=11 each group). Regarding strength training, the subjects were training for at least 6 months, with exercise sessions composed of a brief warmup of 5 min of moderate treadmill, 3 sets for each type of strength exercise with 1 min rest between sets and 2 min rest between exercises. The training load was systematically adapted to keep the maximal possible repetition per set between 10 and 15 repetitions. The strength training program consisted of exercises for all major muscle groups. Exercises to strengthen the upper body included bench press and inclined bench press (pectoralis), shoulder press (trapezius, latissimus dorsi), pull downs (back muscles), biceps curls, triceps extensions, and exercises for abdominal muscles (sit-ups). Lower body exercises included leg press (quadriceps femoris) and squat (thighs, hips, and buttocks, quadriceps femoris muscle).

During the evaluation, subjects were instructed to avoid alcohol and caffeinated beverages for the preceding 12 h and were invited to arrive at the laboratory between 08.30 and 12.30. Upon arrival, the subjects underwent routine clinical examination. Heart rate (HR), systolic arterial blood pressure (SAP), and diastolic arterial blood pressure (DAP) were measured three times at rest, after 15 min in sitting position. Casual AP was measured by the same researcher with a mercury sphygmomanometer and appropriately sized cuffs. The means of the 3 readings were used for analysis.

The RR interval, consecutive heartbeats between each cardiac cycle, were continuously recorded during the whole procedure (15 min) using the Polar S-810i monitor (Finland). HR was determined by the mean value obtained in the RR recording. The spectrum resulting from the Fast Fourier Transforms (FFT) modeling was derived from all the data found in the recorded signal; it included the entire signal variance, regardless of whether its frequency components displayed specific spectral peaks or as non-peak broadband powers (12). RR interval variability was evaluated in frequency domains. Spectral power for low (LF; 0.04–0.15 Hz) and high (HF; 0.15–0.4 Hz) frequency bands was calculated by means of power spectrum density integration within each frequency bandwidth, using a customized routine (MATLAB 6.0) (3).

The power of the sample was calculated *a posteriori* considering the variances of the groups for the variance of RR interval, and the LF/HF ratio obtained a β of 0.99 for both parameters. Data are reported as means and SE. Levene's test was used to assess variance homogeneity. Statistical tests included Student's *t*-test and two-way ANOVA, followed by the *post hoc* of Student-Newmann-Keuls test. A P value <0.05 was considered significant.

## Results

No difference was observed in age, body mass index, weight, and height. Hemodynamic cardiovascular measurements indicated that SAP, DAP, and HR were similar among groups (Table 1). There was no difference in the duration (A-ON: 38±11 and A-OH: 41±12 months) or frequency (A-ON: 4.0±0.3 and A-OH: 3.5±0.2 times/week) of strength training between active groups.

**Table 1.** Baseline demographics and hemodynamics in studied groups.

	S-ON (n=11)	A-ON (n=11)	S-OH (n=11)	A-OH (n=11)
Age (years)	22±1	26±1	23±1	27±2
Weight (kg)	78±5	81±4	80±5	76±3
Height (cm)	179±3	175±2	175±2	174±1
BMI (kg/m^2^)	24±1	26±1	26±1	25±1
SAP (mmHg)	110±2	119±1	111±3	118±1
DAP (mmHg)	76±3	73±1	77±2	69±1
HR (bpm)	72±2	75±1	75±2	74±1

Data are reported as means ± SE. S-ON: sedentary offspring of normotensive parents; A-ON: active offspring of normotensive parents; S-OH: sedentary offspring of hypertensive parents; A-OH: active offspring of hypertensive parents. BMI: body mass index; SAP: systolic arterial pressure; DAP: diastolic arterial pressure; HR: heart rate.

**Figure 1.**
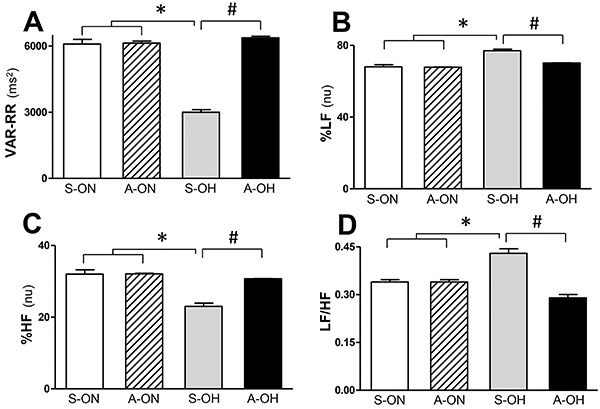
*A*, Variance of RR interval (VAR-RR); *B*, Percentage of LF (low-frequency band, 0.04 Hz and 0.15 Hz) and *C*, HF (high-frequency band, 0.15 Hz and 0.4 Hz); *D*, LF/HF ratio in sedentary offspring of normotensive parents (S-ON), active offspring of normotensive parents (A-ON), sedentary offspring of hypertensive parents (S-OH), and active offspring of hypertensive parents (A-OH). Data are reported as means±SE. *P<0.05 S-OH *vs* S-ON and A-ON; ^#^P<0.05 A-OH *vs* S-OH (ANOVA).

HRV was analyzed by standard deviation and total variance (Figure 1A) of the RR interval and was found to be reduced in the sedentary offspring of hypertensives (S-OH: 57±3 ms and 2998±395 ms^2^) compared to the groups of offspring of normotensives (S-ON: 74±4 ms and 6089±705 ms^2^ and A-ON: 79±2 ms 6133±316 ms^2^). On the other hand, the active offspring of hypertensives (A-OH: 76±1 ms and 6373±226 ms^2^) showed increased standard deviation and variance (Figure 1A) of RR interval, and these variables were normalized when compared to groups of offspring of normotensives.

In the frequency domain analysis of HRV, sedentary offspring of hypertensives presented an increase on cardiac sympathetic modulation (LF-band, S-OH: 77±3 *vs* S-ON: 68±4 and A-ON: 68±0.5%) (Figure 1B), a decrease in parasympathetic modulation (HF-band, S-OH: 23±3 *vs* S-ON: 32±4 and A-ON: 32±0.5%) (Figure 1C) along with increased cardiac sympathovagal balance (LF/HF ratio, S-OH: 0.43±0.04 *vs* S-ON: 0.34±0.02 and A-ON: 0.34±0.02) (Figure 1D). In contrast, strength-trained offspring of hypertensives did not demonstrate autonomic dysfunction in both LF and HF bands (A-OH group, LF: 70.2±0.3% and HF: 29.7±0.3%). Additionally, strength-trained offspring of hypertensives presented reduction in cardiac sympathovagal balance in relation to sedentary ones (LF/HF ratio, A-OH: 0.30±0.02 *vs* S-OH group: 0.43±0.04), reaching similar values in the A-OH group compared to the S-ON and A-ON groups (Figure 1D). No statistical difference in HRV was observed between sedentary and active groups of offspring of normotensives.

## Discussion

It is well-established that family history plays a pivotal role in hypertension onset, and cardiovascular autonomic dysfunction has been found to be a risk marker for development of cardiovascular disease. Thus, we aimed in this study to investigate if regular strength training would have a positive impact on the autonomic modulation of the offspring of hypertensives. Two important findings stand out in this study. First, the sedentary offspring of the hypertensive group showed impaired total RR interval variance and cardiac sympathovagal balance (LF/HF ratio), although the groups did not differ with regard to clinical arterial blood pressure. Second, the strength-training offspring of hypertensives presented increased total variance in the RR interval and decreased cardiac sympathovagal balance compared to the sedentary group. These findings suggested that changes in cardiac autonomic modulation preceded changes in arterial blood pressure, and that strength training had a positive impact on individuals genetically predisposed to hypertension.

Family history of hypertension is a key non-modifiable risk factor for the onset of hypertension, as it increases this risk by 1.4 (4). It should be mentioned that sympathetic nerve activity has been regarded as a major etiologic factor in human essential hypertension (13). Thus, assessing the sympathetic activity/modulation is essential for the offspring of hypertensives.

Neural networks represent a mechanism selected by evolution to control physiological homeostasis. There are several techniques used for sympathetic quantification (14); however, HRV provides valid non-invasive indices for overall sympathetic nerve activity (15). In the present study, the sedentary offspring of the hypertensive group showed a reduction of variance of the RR interval along with a significant increase in cardiac sympathetic modulation. This was demonstrated by an increase in the LF band of the RR interval and by an impairment of vagal modulation, which in turn was demonstrated by a reduction in the HF band of the RR interval. These findings corroborate previous studies, which have also shown an impairment in the sympathovagal balance in the offspring of hypertensive parents (3). Despite the impairment in cardiac autonomic modulation, no difference was found among the groups studied with regard to clinical arterial blood pressure. These results indicate that family history may be very useful to identify cardiovascular risk factors and help design targeted interventions.

On the other hand, a physically active lifestyle has been suggested as a non-pharmacological strategy to prevent hypertension (7). The beneficial effects of aerobic exercise training on cardiovascular parameters in humans (8), as well as in experimental models (11) is well-established. However, to our knowledge, our study is the first to assess the effects of a long period of strength training in men with familial history of hypertension. In our study, strength trained offspring of hypertensives did not show the early changes in HRV observed in sedentary ones. Offspring of hypertensives who underwent strength training showed an increase in variance of the RR interval and a reduction in cardiac sympathovagal balance compared to the sedentary offspring of hypertensives. One of the possible explanations is that strength training may induce increase in baroreflex sensitivity and in nitric oxide bioavailability, thus improving vascular and endothelial functions (16,17). Similarly, improvement in cardiovascular autonomic control after a resistance exercise training protocol was observed in studies involving pre-hypertensive (18) and in experimental models of hypertension (11), but these findings were accompanied by reduced levels of basal arterial blood pressure. In the present study, we observed no reduction in clinical arterial blood pressure in physically active offspring of hypertensives, probably because the subjects of this group were normotensive.

Some limitations of this study should be mentioned. First, the small number of participants. Second, only questionnaires were used to determine the family history of hypertension, and offspring studies may require more information about the history of parental hypertension or normotension (19). Third, the auscultation method used for assessing arterial blood pressure is a rather limited procedure compared to invasive methods; however, all safeguards were taken to ensure the reliability of our results.

In conclusion, sedentary offspring of hypertensives, despite unchanged arterial blood pressure, showed reduced HRV, reinforcing the hypothesis that autonomic dysfunction precedes arterial blood pressure increase (20). Moreover, given that changes in HRV have been associated with a higher risk of hypertension onset (4), our results demonstrated that the impairment on HRV observed in sedentary offspring of hypertensives was not observed in strength-trained offspring of hypertensives. Taken together, these findings reinforce the benefits of an active lifestyle on the prevention of early dysfunction associated with onset of hypertension in predisposed populations.
